# Validity and reliability of OIDP and OHIP-14: a survey of Chinese high school students

**DOI:** 10.1186/1472-6831-14-158

**Published:** 2014-12-19

**Authors:** Li Hongxing, Thomas List, Ing-Marie Nilsson, Anders Johansson, Anne Nordrehaug Astrøm

**Affiliations:** Department of Clinical Dentistry – Prosthodontics, University of Bergen, Bergen, Norway; Orofacial pain and Jaw Function, Malmö University, Malmö, Sweden; Specialist Center for Oral Rehabilitation, Norrköping, Sweden; Department of Clinical Dentistry – Prosthodontics, University of Bergen, Bergen, Norway; Department of Clinical Dentistry – Community Dentistry, University of Bergen, Bergen, Norway

**Keywords:** OIDP, OHIP-14, Adolescents, China

## Abstract

**Background:**

To determine the impact of oral diseases on everyday life, measures of oral quality of life are needed. In complementing traditional disease-based measures, they assess the need for oral care to evaluate oral health care programs and management of treatment. To assess the reliability and validity of the Oral Impact of Daily Performance (OIDP) and the short-form Oral Health Impact Profile (OHIP-14) among high school students in Xi’an, the capital of Shanxi province, China.

**Methods:**

Cross-sectional one-stage stratified random cluster sample using high schools as the primary sampling unit. Students completed self-administered questionnaires at school. The survey included the OHIP-14 and OIDP inventories, translated and culturally adapted for China, and global oral health and socio-behavioral measures.

**Results:**

A total of 5,608 students participated in the study, with a 93% response rate (mean age 17.2, SD 0.8, 52% females, 45.3% urban residents).The proportion experiencing at least one impact (at any frequency) during the previous six months was 62.9% for the OHIP-14 and 45.8% for the OIDP. Cronbach’s alpha measured internal consistency at 0.85 for OHIP-14 and 0.75 for OIDP while Cohen’s kappa varied between 0.27 and 0.58 for OHIP-14 items and between 0.23 and 0.65 for OIDP items. Kappa scores for the OHIP-14 and OIDP additive scores were 0.52 and 0.66, respectively. Both measures varied systematically and in the expected direction, with global oral health measures showing criterion validity. The correlation between OIDP and OHIP-14 was r_s_ +0.65. That both measures varied systematically with socio-behavioral factors indicates construct validity.

**Conclusion:**

Both the OIDP and OHIP-14 inventories had reasonable reliability and construct validity in relation to subjective global oral health indicators among adolescents attending high schools in China and thus appear to be useful oral health –related quality of life measures in this context. Overall, the OHIP-14 and OIDP performed equally well, although OHIP-14 had superior content validity due to its sensitivity towards less severe impacts.

## Background

Both in high and low income countries, outcomes in dentistry have relied on clinical measures considered important from a clinician’s point of view [1]. Although informative, the clinical approach has been criticized because it does not consider functional and psychosocial aspects of oral health. Thus, a shift towards patient-important outcomes has been promoted [1–3]. Concerns that clinical measures alone may not be adequate for assessing people’s oral health needs have sparked the development of Oral Health-Related Quality of Life (OHRQoL) measures. Tested in a variety of populations, OHRQoL measures are increasingly used to complement clinical indicators [1–3]. Increased use of OHRQoL measures warrants cross-cultural adaptation of the existing instruments. Efforts are ongoing to translate and adapt OHRQoL measures for use in non-western cultural settings [4, 5].

OHRQoL indices should be simple to use, reliable, valid, precise, sensitive for change, and amenable to statistical analysis. Two indices come close to meeting those criteria: the eight-item Oral Impacts on Daily Performance (OIDP) scale and the 14-item Oral Health Impact Profile (OHIP-14) [6–8]. Both measures are based on the conceptual framework of the World Health Organization’s International Classification of Impairments, Disabilities and Handicaps, (ICIDH) [9], amended for dentistry by Locker [10]. The OIDP focuses on measuring the most severe oral impacts, namely disability and handicap [6]. The OHIP-14 is derived from the original 49-item OHIP questionnaire. It assesses seven dimensions of impact, including functional limitations, pain, psychological discomfort, physical disability, psychological disability, social disability, and handicap [7, 8]. In terms of respondent burden, both the OIDP and OHIP-14 inventories are relatively short and thus suitable for use in population surveys. Both measures seem to perform well using un-weighted, rather than weighted, scores, although the individually sensitive weighting system of the OIDP inventory gives prominence and increased validity to respondent views [11, 12].

The OIDP and its specific child version, the Child OIDP, appear to be applicable to the general younger populations, as found for instance in Great Britain, Tanzania, Uganda, Brazil, and Peru [13–18]. The OHIP-14, one of the most commonly used generic OHRQoL measures, has proved reliable and valid among both young and middle-aged people in Sweden [19], Brazil [20], Scotland [21], New Zealand [22] and Japan [23]. There have been few reports comparing OHIP-14 and OIDP. In a cross-sectional study involving adolescents in Myanmar, both OIDP and OHIP-14 showed reasonably satisfactory psychometric properties [24]. However, OHIP-14 emerged as the superior measure with respect to construct validity in that it discriminated better than the OIDP between groups with impacts and those without [24]. Robinson et al. [25] found similar results comparing OIDP and OHIP-14 among dental attendees in the UK. Baker et al. [26] compared OHIP-14 and OIDP in UK dental patients with xerostomia and found that the OHIP-14 inventory performed better overall than the OIDP. Bernabe et al. [18], reported a moderate level of agreement between the OIDP and OHIP-14 in a sample of Brazilian adolescents, probably due to differences in the scoring systems and content of oral impacts between the two inventories.

Few attempts have been made to evaluate OHRQoL measures among young people from the general population in developing countries [14–17]. Comparisons between OHRQoL measures are even more seldom in non-occidental contexts [24]. The epidemiology of oral health-related quality of life among young people in China, the world’s most populous and second largest country by area, remains unclear. This is notable because young people are a major focus of dental public health care, globally. Moreover, where resources are scarce, patient-based oral health outcomes can help ensure that services are directed at the conditions most likely to negatively impact OHRQoL.

The present study assessed the reliability and validity of two OHRQoL measures in a sample of Chinese high school students: the abbreviated 14-item OHIP (OHIP-14) and the OIDP. These measures were chosen since they are both derived from theory and relatively short and are thus suitable for questionnaire-based population surveys [13]. The study compared the internal consistency reliability, test-retest reliability, and construct validity of the OIDP and OHIP-14 inventories. Construct validity was assessed by evaluating the relationship of each inventory with global oral health ratings, socio-demographic factors, and oral health-related behaviors as well as the association and level of agreement between OIDP and OHIP-14.

## Methods

### Study area

A cross-sectional survey was conducted in 2008 in Xi’an, a sub-provincial city and the capital of Shaanxi Province, a north-central province of China. The Gross Domestic Product (GPD) of Shaanxi Province for 2007 was USD 70.6 billion and USD 1,887 per capita. According to official records, the province included 37.4 million residents at the end of 2007 [27]. At the time of the survey, the province had approximately 963,300 students in high school. This included about 65,000 students enrolled in 153 high schools in Xi’an itself, which consists of six urban and six rural districts.

### Sampling

The study population included students aged 15–19 years attending Grade 2 in 16 high schools in Xi’an. The survey used a proportionate two-stage cluster sampling design with high schools as the primary sampling unit, stratified by urban versus rural schools and province- , city- and normal level schools. In China, schools are ranked according to the quality of teaching provided into province-level schools (level 1), city-level schools (level 2), and normal-level schools (level 3). To obtain a sample of schoolchildren of mixed socio-economic background, the study randomly selected schools from urban and rural areas in Xi’an, which included a total of 146 high schools (49 urban and 97 rural). Our study stratified the population of schools into urban and rural schools and into “good” (including level 1 schools) and “normal” (including level 2 and level 3 schools) schools. Schools were randomly selected from 28 “good” schools and 69 “normal” schools in the rural and from 8 “good” schools and 41 “normal” schools in the urban areas. Because this study included several outcomes, we calculated the necessary sample size separately for each outcome and adopted the largest sample size required. Calculations showed a sample size of 3,606 to be satisfactory for two-sided tests, assuming the prevalence of oral impacts to be 0.40 and 0.50 in children reporting good versus bad general health with a significance level of 5%, power of 90%, and a design factor of 2 [28]. The first stage selected 3 “good” and 7 “normal” schools in the rural areas (10 rural schools, k = 0.2) and 1 “good” and 5 “normal” schools in the urban (6 urban schools, k = 0.1) by systematic random sampling. The second stage included all Grade 2 students in the selected schools, n = 5,940 pupils.

A total of 5,608 (52% females, mean age 17.2 years, SD 0.8) students completed self-administered questionnaires at school (participation rate 93%). The study excluded students who were physically unable to participate. Each student and their guardian signed informed-consent forms. In cases in which guardians were absent, the classroom teacher signed on their behalf. Only consenting subjects were included in the study and none of the students invited to participate were ill, had a history of psychiatric problems, or were disabled. Ethical clearance was obtained from all relevant persons, authorities and committees. The Health Committee of Shaanxi Province, China and the Regional Ethical Committee (REK VEST) in Norway provided ethical approval.

### Translation and adaptation of the OIDP and OHIP-14 inventories

Professionals fluent in Chinese and English translated the structured questionnaire schedule, including the OIDP and OHIP-14 inventories, from English into the local language of Xi’an. The questionnaire was then back-translated into English by two independent translators. A group of dental professionals reviewed the Chinese version of the questionnaires for semantic, experiential, and conceptual equivalence with the source versions [6–8], considering sensitivity to culture and appropriate word choice. The questionnaire was then pilot tested in a convenience sample of high school students. This test confirmed the feasibility of the methodology and helped to determine the time necessary to complete the questionnaire. In accordance with previous studies applying the OIDP and OHIP-14 inventories to young people [13–22], the participants of this study were able to respond to the questions without the aid of pictures and had no difficulty understanding either the content of the questionnaire or any specific words.

### Measures

The students filled out the OIDP and OHIP-14 frequency questionnaires at school. The OIDP evaluates difficulty performing eight activities of daily life due to dental problems affecting eating, speaking, cleaning the mouth, sleeping, smiling, studying, emotions, and maintaining social contact. Each item received scores 0–4 where 0 = never, 1 = less than once a month, 2 = once or twice a month, 3 = once or twice a week, 4 = every day/nearly every day [5]. Two constructions expressed total OIDP scores, the additive score (ADD) and the simple count (SC). The ADD score (range 0–24) combines the 8 performance scores, 0–4. The SC score (range 0–8) first dichotomizes the frequency items, where 1 = affected (corresponding to original scores 1–4) and 0 = unaffected (including original score 0), before adding them together.

The OHIP-14 items refer to seven dimensions of impact, with participants asked to disclose the frequency of impact on Likert scales where 0 = never, 1 = hardly ever, 2 = occasionally, 3 = fairly often, and 4 = very often. The total OHIP-14 scores are also adjusted into ADD and SC scores, with ADD combining the originally scored 14 items (range 0–56) and SC (range 0–14) combining dichotomized frequency items, with 1 = affected (original categories 1–4) and 0 = unaffected (including the original category 0) [6, 7]. *Parents’ level of education* was originally scored from 1 = no education to 6 = college or university education. Analysis recoded these variables (mother’s and father’s education) into 0 = low education (up to nine years of schooling) and 1 = high education (more than nine years of schooling). One item assessed *economic wealth,* asking “What do you think about your family’s economic situation compared to other families in the area you live?” The four original categories for this item were dichotomized into 1 = poor economic wealth and 2 = moderate to good economic wealth. Other items assessed *family wealth* as an indicator of socio-economic status according to a standard approach in equity analysis [29]. These items asked about durable household assets indicative of family wealth (i.e. washing machines, showers, DVD players, TVs, computers, air conditioners, refrigerators, microwave ovens, mobiles, motorcycles, and cars). These items were either 1 = available and in working condition, or 0 = not available or not in working condition. The assets were analyzed using principal components analysis (PCA) ranging from the 1^st^ poorest quartile to the least poor 4^th^ quartile. *Self- reported oral health status, satisfaction with teeth/mouth, and self-rated health status* were coded on four-point Likert scales which analysis then recoded further into dummy variables of 0 = good/satisfied, and 1 = bad/dissatisfied. *Frequency of chocolate consumption* was originally assessed on a scale ranging from 1 = more than once a day to 4 = seldom or never. Analysis dichotomized this into 1 = categories 1 and 2, and 0 = categories 3 and 4. *Dental attendance during the past 2 years* was recoded as 1 = yes, and 2 = no.

### Statistical analyses

Data analysis used IBM SPSS Statistics version 21 (SPSS Inc., Chicago, IL, USA). To adjust for the effect of the cluster design, data were reanalyzed using STATA 13.0 with survey command. The P-value for statistical significance was 0.05. Cohen’s kappa and Intra class correlation coefficient (ICC) assessed test-retest reliability for 194 students at a time interval of one week. Cronbach’s alpha assessed internal consistency reliability. We examined construct validity by comparing the OHIP-14 and OIDP scores of groups that differed in their global measures of oral health and health status and by estimating the correlation and agreement between the two OHRQoL measures. Moreover, we assessed construct validity by estimating differences in OHIP-14 and OIDP between groups according to socio-economic and behavioral characteristics. For the purpose of cross-tabulation and multiple variable logistic regression analysis, the OIDP SC score (0–8) and the OHIP-14 SC score (0–14) were dichotomized to produce the categories 0 = no daily performance affected, and 1 = at least one daily performance affected. The distribution of the OIDP SC and OHIP-14 SC scores supported this cut-off point. We then reanalyzed the data using Poisson regression with robust variance estimation in STATA 13.0.

## Results

Table 1 summarizes the predictor variables and the number of subjects by categories. Rural students were older, had parents with lower education, and belonged more often to the poorest quartile of family wealth than did their urban counterparts.

**Table 1:** **Frequency distribution of adolescents’ socio-demographic and behavioral factors by urban and rural place of residence**

	Urban (N = 2486) ^a^% (n)	Rural (N = 3032) ^a^% (n)
Male	47.0 (1168)	48.5 (1472)
Female	53.0 (1318)	51.5 (1560)
Young (aged)	66.8 (1678)	36.0 (1100)
Older (aged)	33.2 (834)	64.0 (1952)**
Father’s education: at most 9 yr	24.9 (573)	58.3 (1679)**
Father’s education: above 9 yr	75.1 (1726)	41.7 (1200)
Mother’s education: at most 9 yr	30.6 (700)	65.6 (1881)**
Mother’s education: above 9 yr	69.4 (1585)	34.3 (983)
Living at schools: no	71.5 (1775)	15.6 (473)**
Living at schools: yes	28.5 (709)	84.4 (2557)
Economic wealth: poor	5.6 (139)	16.7 (506)**
Economic wealth: moderate to good	94.4 (2336)	83.3 (2517)
Housekeeper: yes	8.0 (196)	1.8 (55)**
Housekeeper : no	92.0 (2264)	98.2 (2925)
Family wealth: least poor: 1^st^ quartile	62.9 (1483)	9.4 (277)
Family wealth: 2^nd^ quartile	23.6 (556)	20.0 (587)
Family wealth: 3^rd^ quartile	9.6 (227)	38.0 (1114)
Family wealth; most poor 4^th^ quartile	3.9 (93)	32.6 (957)**
Brushing teeth: less than twice a day	39.1 (972)	59.3 (1794)
Brushing teeth: twice a day or more	60.9 (1513)	40.7 (1232) **
Chocolate: seldom	36.9 (916)	49.3 (1491)
Chocolate: at least several times a week	63.1 (1567)	50.7 (1534) **
Dental attendance last 2 yr: yes	58.4 (1439)	29.4 (2130)
Dental attendance last 2 yr: no	41.6 (1026)	70.6 (2401) **
Smoking: no	96.7 (2401)	89.4 (2696)
Smoking: yes	3.3 (83)	10.6 (320) **

^a^The total numbers in the different categories do not add up to 5608 participants due to missing values.

**p < 0.001.

Using both the additive (ADD) and simple count (SC) scoring systems, the OIDP showed that 38.1% of urban- and 52.2% of rural students reported at least one area of daily life activity to be affected (p < 0.001) (Table 2). The corresponding figures for the OHIP-14 were 55.6% and 68.9% (p < 0.001). Mean OHIP-14 ADD scores amounted to 2.0 (SD 3.4) in urban and 3.0 (SD 4.3) in rural residents (p < 0.001). The corresponding figures for mean OIDP scores were 0.9 (SD 1.9) and 1.5 (SD 2.5) (p < 0.001). Impact on eating was the most frequently reported OIDP in urban areas (19.2%) followed by impact on cleaning teeth (12.8%) and smiling (15.5%). The most commonly reported impacts in rural areas were eating (26.5%) followed by smiling (25.7%) and cleaning teeth (2.6%). According to OHIP-14, the most common problem among urban and rural residents was pain (38.0% and 44.9%), followed by self-consciousness (23.6% and 29.6%), eating (20.3% and 26.8%) and embarrassment (14.0% and 21.6%).

**Table 2:** **Percentage distribution of OIDP and OHIP-14 performance items and total prevalence scores for urban and rural residents**

OIDP			OHIP-14		
Items	Urban % (n)	Rural % (n)	Items	Urban % (n)	Rural % (n)
Eating	19.2 (484)	26.5 (810)**	Articulation	5.4 (136)	9.4 (288)**
Speaking	5.4 (135)	9.5 (290)**	Sense of taste	9.3 (236)	18.9 (578)**
Cleaning teeth	12.8 (322)	21.6 (657)**	Pain	38.0 (958)	44.9 (1369)**
Sleeping/ Relaxing	7.4 (184)	14.2 (433)**	Eating	20.3 (512)	26.8 (820)**
Smiling	15.5 (391)	25.7 (783)**	Self-conscious	23.6 (597)	29.6 (904)**
Emotional	5.0 (1|26)	8.7 (266)**	Felt tense	9.1 (230)	12.8 (391)**
School work	5.7 (144)	14.2 (432)**	Diet unsatisfactory	13.2 (333)	22.0 (673)**
Social contact	2.0 (50)	2.9 (88)*	Interrupt meals	7.0 (178)	9.6 (295)**
OIDP > 0	38.1 (949)	52.2(1573)**	Relax	8.8 (222)	15.9 (487)**
Mean OIDP ADD	0.9 (1.9)	1.5 (2.5)**	Embarrassed	14.0 (353)	21.6 (661)**
			Irritable	6.4 (163)	15.4 (470)**
			Usual work	5.2 (132)	6.8 (207)*
			Less satisfied	7.1 (179)	13.1 (401)**
			Unable to function	6.3 (159)	11.1 (339)**
			OHIP-14 > 0	55.6 (1383)	68.9 (2073)**
			Mean OHIP-14 ADD	2.0 (3.4)	3.0 (4.3)**

**p < 0.001, *p < 0.05.

### Reliability

All participants completed the OIDP and OHIP-14 frequency inventories, suggesting that the inventories were readable and interpretable, thus providing support to face validity. The percentages of missing responses varied from 0.6%-1% across the single OIDP items and from 0.2%-0.6% across the single OHIP-14 items. No method to substitute missing values was performed in this study. Internal consistency reliability (standardized item alpha) was 0.75 for the OIDP and 0.85 for the OHIP-14. The corrected item total correlation (i.e. the correlation between each item and the total score omitted for that item) ranged from 0.40 (embarrassed) to 0.63 (diet unsatisfactory) for the OHIP-14 and from 0.40 (social contact) to 0.56 (emotional state) for the OIDP with a minimum level of 0.20 required to include an item in the scale [30]. The Cronbach’s alpha decreased when any one item was deleted from the scales. This was more systematically true for the OIDP than for the OHIP-14 score. Test-retest reliability in terms of Cohen’s kappa varied between 0.27 and 0.58 for the single dichotomized OHIP-14 items and between 0.23 and 0.65 for the single dichotomized OIDP items. Kappa scores for the dichotomized OHIP-14 ADD and OIDP ADD scores were 0.52 and 0.66, respectively. ICC scores for the continuous OHIP-14 ADD and OIDP ADD scores were 0.84 (95% CI 0.79-0.88) and 0.86 (95% CI 0.82-0.89),respectively.

### Construct validity

Criterion validity was demonstrated in that the OHIP-14 and OIDP scores increased as the status of students’ self-reported oral health, general health, dental appearance, and oral problems changed from healthy to unhealthy. This was shown by the Chi-square test in cross-tabulation analyses and with Spearman’s correlation coefficients using both SC and ADD scores (Table 3). Students not satisfied with their oral health and who rated their tooth status as bad had consistently higher rates of OIDP and OHIP-14 impacts than students who felt satisfied and rated their tooth status as good. Spearman’s correlation for the OIDPADD scores ranged from 0.17 for general health status to 0.34 and 0.32 for perceived state of teeth. Corresponding coefficients for the OHIP-14 ADD scores were from 0.19 for general health to 0.36 and 0.34 for perceived state of teeth. Spearman’s correlation between the total scores of OIDP and OHIP-14 (i.e., derived from both scoring systems) was 0.65 (p < 0.001). The Cohen’s kappa for the OHIP-14 SC and OIDP SC scores was 0.45. A total of 23% reported no impact in the OIDP and at least one impact in the OHIP-14, whereas 6% reported at least one impact in the OIDP but no impact in the OHIP-14.

**Table 3:** **Criterion validity: percentages and number (n) of students having at least one impact according to global oral health measures and the correlation between measures of global oral health and OIDP ADD and OHIP14 ADD scores**

	OIDP SC		OIDP ADD	OHIP-14 SC		OHIP-14 ADD
	% (n)	Rho	Rho	% (n)	Rho	Rho
**State of teeth**						
Good	34.2 (1236)	0.34	0.32	53.2 (1927)	0.36	0.34
Bad	68.3 (1282)**			82.0 (1527)**		
**Satisfied with teeth**						
Yes	37.5 (1508)	0.32	0.32	55.7 (2235)	0.33	0.34
No	68.3 (1011)**			82.7 (1221)**		
**Tooth position**						
Satisfied	37.4 (1337)	0.25	0.26	56.3 (2006)	0.22	0.22
Dissatisfied	61.3 (1181)**			75.1 (1447)**	0.25	0.25
**Tooth appearance**						
Satisfied	36.1 (1253)	0.27	0.28	55.4 (1917)		
Dissatisfied	62.4 (1265)**			75.8 (1536)**		
**Tooth color**						
Satisfied	36.2 (1121)	0.24	0.24	53.7 (1657)	0.26	0.26
Dissatisfied,	58.1 (1397)**			74.8 (1797)**		
**General Health**						
Good	42.5 (1879)	0.17	0.17	60.1 (2654)	0.19	0.19
Bad	59.2 (641)**			74.6 (802)**		

**p < 0.001.

Cross tabulations and Spearman’s rho.

Standardized logistic regression- and Poisson regression analyses explored socio-demographic and behavioral covariates of the total prevalence and extent of oral health-related quality of life as assessed by the OIDP and OHIP-14 (Table 4). Socio-demographic variables, entered in the first step, provided model summaries of Nagelkerke’s R^2^ = .044 for both the OIDP SC and OHIP-14 SC models. By entering the oral health behavioral variables in the second step, the model summaries of Nagelkerke’s R^2^ increased to 0.085 for the OHIP-14 model and to 0.082 for the OIDP model. In the final OHIP-14 model, the statistically significant predictors were gender, district of residence, mother’s education, family wealth index, brushing, chocolate consumption, smoking, and dental attendance in the last two years. The corresponding odds ratios were respectively, 1.6, 1.6, 0.8, 1.4, 0.8, 1.4, 1.7 and 0.4. In the final OIDP model, the significant predictors were: gender OR = 1.5, residence OR = 1.3, family wealth OR = 1.4, brushing OR = 0.8, chocolate consumption OR = 1.2, smoking OR = 1.2, and dental attendance OR = 0.5. When data were reanalyzed using STATA 13.0 and Poisson regression with robust variance estimates, the results were essentially unchanged.

**Table 4:** **Construct validity: percentages and (n) of students having at least one oral impact by socio-demographic characteristics and oral health-related behaviors and OIDP and OHIP14 regressed on socio-demographic and behavioral factors**

	OIDP unadjusted % (n)	Adjusted OR (95% CI)	OHIP-14 unadjusted % (n)	Adjusted OR (95% CI)
Male	41.3 (1083)	1	58.4 (1533)	1
Female	49.8 (1426)**	1.6 (1.4-1.7)	67.2 (1915)**	1.6 (1.4-1.8)
Younger	42.8 (1173)	1	59.7 (1633)	1
Older	48.8 (1336)**	1.1 (0.9-1.2)	66.3 (1811)**	1.1 (0.9-1.2)
Urban residence	38.1 (949)	1	55.6 (1383)	1
Rural residence	52.2 (1573)**	1.3 (1.1-1.5)	68.9 (2073)**	1.6 (1.4-1.9)
Fathers education: Low	50.1 (1131)	1	67.2 (1756)	1
Father’s education: higher	43.0 (1251)**	0.9 (0.8-1.1)	60.4 (1756)**	0.9 (0.8-1.1)
Mother’s education: lower	50.3 (1295)	1	67.6 (1734)	1
Mother’s education: higher	42.0 (1078)**	0.8 (0.7-1.0)	59.5 (1525)**	0.8 (0.7-0.9)
Living in school: no	39.5 (889)	1	58.0 (1299)	1
Living in school : yes	50.2 (1635)*’	1.1 (0.9-1.3)	66.2 (2158)**	0.9 (0.8-1.2)
Wealth: Poor	53.3 (344)	1	69.0 (445)	1
Wealth: good	44.8 (2173)**	0.9 (0.7-1.2)	62.2 (3008)**	0.8 (0.7-1.0)
Family wealth: Least poor	37.1 (518)	1	54.5 (758)	1
“	41.5 (560)	1.1 (0.9-1.4)	61.4 (824)	1.2 (0.9-1.3)
“	49.2 (670)	1.3 (1.1-1.6)	65.3( 893)	1.1 (0.9-1.4)
Family wealth: Poorest	55.4 (754)**	1.4 (1.3-2.1)	70.6 (960)**	1.4 (1.1-1.7)
Brushing teeth: less than twice a day	47.9 (1321)	1	64.9 (1791)	1
Brushing teeth: twice a day or more	43.7 (1200)**	0.8 (0.7-0.9)	60.9 (1666)	0.8 (0.7-0.9)
Chocolate: seldom	44.1 (1061)	1	59.2 (1425)	1
Chocolate: at least several times a week	47.1 (1459)**	1.2 (1.0-1.3)	65.8 (2029)**	1.4 (1.2-1.6)
Dental attendance last 2 year: yes	55.8 (825)	1	71.9 (1060)	1
Dental attendance last 2 year: no	41.9 (1620)**	0.5 (0.3-0.5)	59.5 (2295)**	0.4 (0.4-0.5)
Smoking: no	45.1 (2055)	1	62.0 (2812)	1
Smoking: yes	48.5 (457) ns	1.2 (1.0-1.4)	67.3 (637)*	1.7 (1.2-1.6)

**p < 0.001, *p < 0.05, ns not statistically significant.

Odds ratios (OR) and 95% confidence interval (CI).

## Discussion

This study compared for the first time a Chinese version of the OIDP and OHIP-14 inventory in a population of adolescent students in Xi’an province, China. Whereas the OIDP has not previously been evaluated in this socio-cultural context, the applicability of the original OHIP-49 and two abbreviated OHIP-14 instruments have been tested in face-to-face interviews with the elderly population and recently among middle aged stroke patients in Hong Kong [31, 32]. Since health is a dynamic state, application of the OHIP-14 among school-going adolescents in China required reestablishment of its psychometric properties when using self-administered questionnaires in accordance with the data collection of the original English OHIP-14 version [33].

The Chinese versions of the OIDP and OHIP-14 appeared to be valid and reliable with psychometric properties similar to their original English versions [6–8]. Moreover, the Chinese version of the OHIP-14 had psychometric properties similar to the abbreviated OHIP versions that were derived for use among elderly in Hong Kong (OHIP-14 original and OHIP-14 Chinese version) [31]. Consistent with a previous study of Brazilian adolescents, this study revealed a moderate level of agreement between the OHIP-14 and OIDP (kappa value 0.45), reflecting differences in content validity [17]. Such moderate agreement may reflect variation in scope, with OHIP-14 assessing oral impacts on all levels, in accordance with the model by Locker [10], while OIDP emphasizes the most severe impacts only: the levels of handicap and disability. The strength of the correlation coefficient between the two inventories (Spearman’s rho 0.65) provided support for their common theoretical origin [9, 10]. The correlation coefficient observed in this study is stronger than that of 0.40 reported by Soe et al. [24] among Myanmar adolescents, but agrees with what was reported by Robinson et al. [25] investigating dental attendees in UK. In general, OIDP and OHIP-14 performed almost equally well among the Chinese school-going adolescents investigated in this study. This indicates that the total burden on participants (additive scores) was as important in this young population as was the number of areas affected (simple count scores).

All Cronbach’s alpha values observed met Nunnally’s standard of 0.70 for appropriate internal consistency in studies involving group comparisons [30]. These figures compare favorably with those obtained in other studies involving young people from high and low income countries [13–19]. In the present study, agreement in terms of kappa values was 0.52 for the presence of an impact as detected by OHIP-14 and 0.66 for OIDP. This corresponds to the test-retest results reported in the study of Myanmar adolescents [24]. Our finding indicates acceptable test-retest reliability, although the kappa value for the presence of an impact denoted moderate agreement for OHIP-14 and good agreement for OIDP [30].

Cultural issues, in particular language, might cause problems with validity [33]. Although no approach guarantees cross-cultural equivalence, the Chinese version of the OIDP and OHIP-14 seem to preserve the overall concepts of their corresponding English versions and do not differ in the sequence of questions, Likert scale, or recall memory period (six months) used. Previous experience regarding the usability of the OHIP inventory in its original- and abbreviated versions in personal interviews among the elderly, as well as recent self-administered questionnaires among school going adolescents, support the cross-cultural equivalence of this inventory. Completion rates for the OIDP (missing items varied from 0.2-1%) and the OHIP-14 (missing items varied from 0.2-0.6%) were acceptable, adding support to the face validity of both measures. There was no indication from the reference groups of academics, or from the pilot study among adolescents, that the relevance of any of the items was low in the context of Chinese school-going adolescents. This suggests that the Chinese high school students were capable of fully understanding the translated version without altering the meaning of the questions and that the Chinese and English versions of the OHIP-14 and OIDP inventories are comparable.

Both measures had significant validity in that they varied systematically, equally strongly, and in the expected direction with global oral health measures (Table 3). Thus, independent of the scoring system, the OIDP and OHIP-14 indicated (to the same degree) lower levels of oral impacts when self-perceived oral health was better. This similarity in performance agrees with the results reported by Baker et al. [26]. It disagrees, however, with the results of Soe et al. [24] and Robinson et al. [25] who reported that the strength of the associations with oral health ratings were weaker for the OIDP than for the OHIP-14. Since the present study considered extent scores of OIDP only, it disagrees with recent findings of Kristapong et al. [34] among Thai adolescents. In that study intensity OIDP scores associated with global oral health ratings whereas extent scores did not. The extent of oral impacts is calculated as a simple count scores (OIDPSC), whereas intensity scores calculate the percentage of respondents with impacts [6]. As in this study, clinical measures have traditionally been excluded from previous validations of the OIDP instrument [6]. The rationale behind the decision to omit clinical variables derives from the conceptual distinction between health and disease [6, 9].

The OIDP and OHIP-14 scores were applicable across age and gender, with females and older students being most likely to report any impact (Table 4). Locker and Miller [35] found younger Canadians to be as likely as their older counterparts to report impacts of oral disorders. Similar findings have been reported among students in Tanzania [14]. Reports have shown that women perceive more negative impacts than do men, suggesting gender differences in their life-course influences [22, 33]. The higher prevalence of impacts reported by disadvantaged students (rural residents from the poorest family wealth category and having mothers with lower education) is probably partly due to material and social deprivation [36]. Greater frequency of smoking and sugar intake seem to imply less favorable students’ OIDP and OHIP-14 ratings. Moreover, the better the brushing and dental attendance ratings the more favorable the oral quality of life ratings. This is consistent with findings among Ugandan adolescents as reported by Åstrøm and Okullo [16] as well as with studies conducted elsewhere in non-occidental cultural contexts [14, 15].

Relatively high proportions of students reported being affected by an oral impact during the six months preceding the survey both for OIDP (45%) and OHIP 14 (62%). The higher number of impacts found with OHIP-14 compared to OIDP could be due to the greater number of items and to different content of the questions included (24, 25). Contrary to the OHIP-14, with its designed sensitivity to less severe impacts of oral condition, OIDP concentrates only on the most severe impacts and may thus be less sensitive in younger populations with lower levels of oral disease. Other studies have reported higher proportions of impacts using OHIP-14 than for OIDP [17, 24, 25]. Eating was the most commonly reported OIDP impact, affecting about 19% of urban and 26% of rural residents using both OIDP and OHIP-14 [13–19, 36]. In contrast, self-consciousness was the most frequently reported OHIP-14 impact (Table 2). Elderly Chinese have reported low frequencies of negative impacts in the psychological, social, and disability domains of OHIP [31]. The present study of young Chinese people found psychological discomfort (self-consciousness) and functional aspects to have the greatest impact in both the OHIP-14 and OIDP. Thus, elderly Chinese may be more likely to accept their oral condition so that oral problems do not hinder their social life as much as for the younger part of the population [31].

The OIDP estimates obtained in this study fell short when compared to the prevalence of OIDP reported among young people elsewhere such as in Uganda [16]. On the other hand, the present OIDP prevalence was higher than that reported among young adults in Tanzania as well as in a nationwide Norwegian study (18%) [14, 36]. The OHIP-14 prevalence and the mean additive OHIP-14 scores compared favorably with those obtained among subjects of the same age in Myanmar [24]. It fell short, however, of the corresponding estimate for dental attendees in UK [22]. The observed variety in prevalence of impacts using identical OHRQoL instruments might be due to differing perceptions of oral health in different populations or to reporting biases. Notably, comparison of prevalence estimates across surveys should be done with caution. Many previous analyses have selected the number of impacts at the frequency of “fairly often” or more with OHIP-14 and at “once a week” or more with the OIDP. Such a high threshold for prevalence was not suitable in the present study because of the skewed distribution of impacts. Thus, in accordance with McGrath and Bedi [37] our study selected the criterion “having impact at any frequency” (all categories included regardless of their frequency) to capture those subjects experiencing only a single impact. Such differences in the use of the instruments may explain variation in levels of impacts across various populations.

Some additional limitations should be acknowledged when interpreting the results. Since regression analyses did not adjust for clinical measures of oral diseases, it is uncertain whether or not the social gradient observed is related to various levels of oral diseases. Moreover, without the possibility of confirming any causal relationship between socio-behavioral factors and OHRQoL with a cross sectional design, the present findings strongly suggest that perceived oral health status is shaped by lifestyles and prevailing social circumstances. The accuracy of reporting perceived impairments in population based studies may be limited. Another caveat may be the inventories using a 6 month recall period relying on self- reports which implies they can be prone to recall bias.

## Conclusion

Both the OIDP and OHIP-14 inventories had reasonable reliability and construct validity in relation to subjective global oral health indicators among adolescents attending high schools in China and thus appear to be useful OHRQoL measures in this context. Overall, the OHIP-14 and OIDP performed equally well, although OHIP-14 had superior content validity due to its sensitivity towards less severe impacts.

## Authors’ information

LH: PhD candidate, University of Bergen, Norway. TL: Professor Oral Facial pain and jaw function, Specialist center for oral rehabilitation, Norrkøping, Sweden, IMN: Clinical consultant, Specialist Center for Oral Rehabilitation, Norrkøping, Sweden. AJ: Professor, University of Bergen, Norway. ANÅ: professor, University of Bergen, Norway.
